# MiR-2284b regulation of α-s1 casein synthesis in mammary epithelial cells of dairy goats

**DOI:** 10.1080/10495398.2024.2334725

**Published:** 2024-04-16

**Authors:** Jinxing Hou, Wenfei Li, Xiaolong Xu, Ao Sun, Ganggang Xu, Zefang Cheng, Haoyuan Zhang, Xiaopeng An

**Affiliations:** a College of Animal Engineering, Yangling Vocational & Technical College, Yangling, Shaanxi, P.R. China; b College of Animal Science and Technology, Northwest A&F University, Yangling, Shaanxi, P.R. China

**Keywords:** miR-2284b, αs1-casein, goat mammary epithelial cells

## Abstract

The lactation character of dairy goats is the most important characteristic, and milk protein is an important index to evaluate milk quality. Casein accounts for more than 80% of the total milk protein in goat milk and is the main component of milk protein. Using GMECs (goat mammary epithelial cells) as the research object, the CHECK2 vector of the CSN1S1 gene and the overexpression vector of pcDNA 3.1 were constructed, and the mimics of miR-2284b and the interfering RNA of CSN1S1 were synthesized. Using PCR, RT–qPCR, a dual luciferase activity detection system, EdU, CCK8, cell apoptosis detection and ELISA detection, we explored the regulatory mechanism and molecular mechanism of miR-2284b regulation of αs1-casein synthesis in GMECs. miR-2284b negatively regulates proliferation and apoptosis of GMECs and αs1-casein synthesis. Two new gene sequences of CSN1S1 were discovered. CSN1S1-1/-2 promoted the proliferation of GMECs and inhibited cell apoptosis. However, it had no effect on αs1-casein synthesis. MiR-2284b negatively regulates αs1-casein synthesis in GMECs by inhibiting the CSN1S1 gene. These results all indicated that miR-2284b could regulate αs1-casein synthesis, thus playing a theoretical guiding role in the future breeding process of dairy goats and accelerating the development of dairy goat breeding.

## Introduction

Worldwide, mainly dairy cows, goats, sheep, buffalo and camels are raised in the dairy industry.^1^ Farming of dairy goats has increased dramatically since the 1960s due to changes in human incomes and taste preferences for dairy products.^2^ Compared with the milk industry, the goat milk industry has its own unique advantages: at the nutritional level, the protein composition of goat milk does not easily produce allergic reactions, which solves the problem of lactose intolerance caused by drinking milk. The fat balls in goat milk are smaller, easier to digest, and more nutritious, which causes goat milk to have competitive value in quality.^3^

The udder of a goat is located in the groin region. The dorsal side appears as a single glandular mass and is divided in two by a well-defined intermediate breast. Each half is made up of a mammary gland and nipple. The lateral surface is raised, and the medial surface is flattened. Mammalian mammary glands are divided into parenchyma and stroma. The mammary lobe is the parenchymal part of the mammary gland, which originates from the ectoderm of the embryo.^4^ For many years, it has been assumed that there is a positive correlation between mammary gland size and milk production in dairy goats.^5^ From a macroscopic perspective, the udder weight of dairy goats does not change until the 120th day of pregnancy. In general, udder growth in dairy goats occurs between the last 30 days of gestation and the first 10 days of lactation. During pregnancy and lactation and during the first 15 days of pregnancy, the composition of breast tissue changes. There is a decrease in the proportion of parenchymal adipose tissue and an increase in fluid-rich tissue. Thereafter, the composition of the mammary glands remain unchanged during late gestation and throughout lactation. The changes in breast tissue composition are directly related to increased milk secretion and fluid accumulation in the breast, suggesting that breast tissue increases exponentially during peak lactation.^6^ From the microscopic point of view, the mammary gland structure of ruminants is basically formed in the embryonic stage.^7^ The mammary duct system is formed in adolescent dairy goats, but at this time, the mammary glands are basically not developed. In the early stage of pregnancy, the breast ducts grow a large number of branches, breast epithelial cells proliferate rapidly, and blood flow in the breast increases.^8^ During the second trimester of pregnancy, mammary epithelial cells continue to grow rapidly, and a large number of different shapes and sizes of acini begin to appear in the mammary glands. In the later stage of pregnancy, the acini are fully developed, the number of organelles inside the acinar epithelial cells begins to become abundant, and the mammary glands begin to secrete milk. Throughout pregnancy, a large number of fat droplets exist in the top membrane of the mammary epithelial cells, as the lactating parenchyma of the breast tissue replaces part of the interstitium.^9^ Lactating mammary epithelial cells show columnar morphology, the mammary acini are very large and irregular in shape, surrounded by thin layers of fibroblasts and capillaries, and the acinar cavities are full of secretions.^10^ This demonstrates that the expansion of mammary gland secretory tissue in dairy goats occurs through an increase in the number and size of vesicles during gestation and early lactation, corresponding to a macroscopic increase in mammary gland volume.

In vitro culture of goat mammary epithelial cells (GMECs) can be used to observe the growth and development process of mammary epithelial cells in vitro more fully and carefully, which provides an opportunity to thoroughly study the mammary gland lactation mechanism in vitro. In addition, in vitro culture of mammary gland epithelial cells can become a vector of gene transfection and expression, providing a scientific model for studying the influence of specific genes on the growth and development of the mammary glands of dairy goats.^11^ The mammary glands of dairy goats are composed of different tissues and therefore a variety of cells, mainly epithelial cells, stromal cells, adipocytes, vascular epithelial cells and immune nucleus mammary stem cells. Epithelial cells are divided into two types, namely, acinar epithelial cells and basal epithelial cells.^12^

MicroRNAs are 18–25 nt noncoding short strand RNAs. They exist widely in species from nematodes to *Homo sapiens* and play an important role in gene expression regulation.^13^ The most unique and important role of miRNAs is the formation of miRNA-induced silencing complexes at the target sites of the corresponding 3′ untranslated region (UTR) of mRNA, which can regulate or inhibit mRNA translation. Each miRNA has multiple target-bound miRNAs, and each posttranscriptional product can also be targeted by multiple miRNAs.^14^ Many studies have demonstrated that miRNAs play a role in many processes in vivo, including apoptosis, metabolism, and differentiation.^15^ Luca Falzone collected and analyzed miRNAs related to breast cancer patients, dietary intervention and physical exercise and screened out some miRNAs that may play a role in the development of breast cancer and may have prognostic value in patients receiving combined intervention therapy such as diet and physical activity.^16^ The different binding sites of miRNAs and target genes may affect the expression of their target genes and thus promote the apoptosis of breast cells.^17^

Since miRNAs are related to cell proliferation, apoptosis and signal transduction, many studies have explored the role of miRNAs in mammary gland growth and development and lactation in recent years. Some studies have sequenced miRNAs in sheep during peak lactation and nonpeak lactation and revealed some miRNAs with high expression and low expression during peak lactation, suggesting that miRNAs from different sources may play different roles in different stages of mammary gland development. miRNAs of different lengths may also play a specific role in mammary gland development.^18^ miR-142-5P can promote the metabolism of milk fat in GMECs and promote the synthesis of triglycerides by inhibiting the expression of CTNNB1.^19^ The target genes of the miR-26 family are related to the PI3K-Akt signaling pathway, MAPK signaling pathway and fatty acid biosynthesis pathway. The expression of the miR-26 family directly affects the total fat yield and the contents of short chain, medium chain and long-chain fatty acids in goat milk.^20^ The miR-2284 family is a ruminant-specific miRNA family. Barozai obtained a novel miRNA of the miR-2284 family in sheep based on a homology search through comparative genetics.^21^ Nathan Lawless mentioned in his article that the miR-2284 family is species-specific in the bovine genome and that miRNAs from this family do not appear to have homology in humans or mice and have been shown to be expressed in many bovine immune-related tissues.^22^ Lee J analyzed microRNA expression profiles in whole blood samples of pregnant and nonpregnant lactating Holstein cows under heat stress conditions and found some differences in the expression of miRNAs from the bta-miR-2284 family. The researchers hypothesized that target genes of the miR-2284 family were involved in heat-induced immune responses, but their biological role was not determined at the time.^23^ Further studies have demonstrated the relationship between the miR-2284 family and mammary glands and lactation. After detecting circRNA expression profiles of lactating cows at 90 and 250 days postpartum, it was found that circRNA from CSN1S1 was highly enriched, and these circRNAs were differentially expressed between the two groups of cows. CircRNAs have targets for the microRNA miR-2284 family, suggesting that they may be involved in regulating the expression of casein genes in milk.^24^

MicroRNAs (miRNAs) regulate mammary gland development and lactation function. In preliminary laboratory experiments,^25^ high-throughput sequencing and bioinformatics methods were used to screen out miR-2284b with significant differential expression from the breast tissues of dairy goats in the early lactation and peak lactation periods, and bioinformatics methods were used to predict that miR-2284b has a targeted binding site on the αs1-casein synthesis gene CSN1S1. Therefore, exploring the molecular mechanism of αs1-casein synthesis and further studying the regulatory effect of miR-2284b on αs1-casein synthesis is of great significance for reducing milk allergens and improving the milk quality of dairy goats, and it is helpful and influential to solve problems in the infant dairy industry.

## Materials and methods

### Animals and ethics

Breast tissues of dairy goats were collected from a farm near Northwest A&F University. Healthy three years adult saanen dairy goats (82Kg ± 4Kg) were selected during the lactation period. After breast tissues were collected, they were stored in PBS supplemented with 100 μg/mL penicillin and streptomycin (Solarbio, China, P1400) and transported back to the laboratory in an ice box for treatment. This research was approved by the Institutional Animal Care and Use Committee of Northwest A&F University (permit number: 17-347, data: 2017-10-13) following the recommendation of the Regulations for the Administration of Affairs Concerning Experimental Animals of China. Then, the breast tissues rinsed with PBS were cultured by the tissue block adhesion method, and when monolayer mammary epithelial cells were formed and purified by the enzyme digestion method, pure primary mammary epithelial cells were finally obtained.

### Primary milk goat mammary epithelial cell collection and culture

First, the breast tissues were rinsed with PBS and then cultured with the tissue block adhesion method. After 2–3 days of culture, the breast epithelial cells continuously grew out of the tissue blocks. Finally, the primary breast epithelial cells were purified with an enzyme digestion method. The breast epithelial cell culture medium used was DMEM/F12 (Gibco, Grand Island, USA, 12400-024), 10% fetal bovine serum (Biological Industries, Israel, 04-001-1ACS) and 100 μg/mL penicillin and streptomycin. Then, these cells were incubated with 5% CO_2_ at 37 °C.

### Cell transfection

GMECs were cultured in 6-well plates or 96-well plates to the logarithmic growth stage (70%–80%) and transfected according to Lipofectamine 2000 protocol (Invitrogen, USA, 11668019). According to the miR-2284b primer sequence 5′-ugaaaaguuuucucggguua-3′, in vitro mimics, inhibitors and a NC (negative control) of miR-2284b were synthesized. Inhibitor NC was incubated with Lipofectamine 2000 OPTI-MEMI medium for 5 min, mixed and incubated again for 20 min, and cell culture plates were placed in a 5% CO2 incubator at 37 °C. After 5 h, the culture was changed to complete medium for 48 h.

### Reverse transcription and RT–qPCR

Milk goat mammary epithelial cells were lysed on ice with TRIzol reagent (TaKaRa, China, 740984.50). Forty-eight hours after transfection, RNA was extracted, and cDNA was obtained by reverse transcription. Reverse transcription was performed according to the PrimeScript RT reagent kit protocol (TaKaRa, China, RR037B). The reverse transcription system was 20 µL. The specific test procedures were as follows. (1) A genomic DNA elimination reaction was performed and the reaction system was 10 µL: 5 × gDNA Eraser Buffer (2.0 µL), gDNA Eraser (1.0 µL), total RNA (4.0 µL), RNase free dH_2_O (3.0 µL). After mixing according to the above system, the solution was incubated at room temperature for 5 min. (2) Reverse transcription was performed using the reaction solution from step 1 (10.0 µL), 5 × Prime Script Buffer 2 (for real-time PCR; 4.0 µL), Prime Script RT Enzyme Mix 1 (1.0 µL), RT Prime Mix (1.0 µL), RNase free dH_2_O (4.0 µL). Then, goat miR-2284b and β-actin mRNA sequences were found in NCBI (https://www.ncbi.nlm.nih.gov/). Primer 5.0 software was used to design primers (Table 1). Then, the 20 µL real-time PCR mixture included TB Green ® Premix Ex Taq^TM^ II (10 µL) (TaKaRa, China, RR091B), cDNA (4 µL), primers (1 µL), and free dH_2_O (up to 20 µL), incubated at 98 °C for 10 min, followed by 40 cycles at 98 °C for 15 s, 60 °C for 1 min using the CFX Connect^TM^ Real-Time PCR Detection System (Bio-Rad, CA, USA). Finally, β-actin was used as a reference gene, and the abundance of mRNA expression was calculated using the 2^-ΔΔCt^ method.

**Table 1.** The quantitative primer sequences.

Name	Sequence (5′∼3′)
MiR-2284b	F: CGCGCGTGAAAAGTTTGTTC
	R: AGTGCAGGGTCCGAGGTATT
CSN1S1-1	F: CAACTGAGGATCAAGCCATGGAAG
	R: CTCAGAGGGCACATCTTCCTTT
CSN1S1-2	F: CAACTGAGGATCAAGCCATGGAAG
	R: ACCCAGGTAACGCTCAGAGG

### Cell proliferation and apoptosis

GMECs were transfected for 24 h in 96-well plates, including miR-2284b mimics/miR-2284b inhibitor and NC/inhibitor NC. After 24 h, cell counting kit-8 (CCK-8) (1 µL) (ZETATM life, USA) was used and incubated 24h, and the light absorption intensity of the cell pore at 450 nm wavelength was measured by an enzyme label analyzer (Biotek, Winooski, USA).

Cells were deposited in 96-well plates and transfected into pc-CSN1S1-1/2, pc-CSN1S1-1/2 + miR-2284b mimics, si-CSN1S1, si-CSN1S1 + miR-2284b inhibitor, NC and empty vector pcDNA3.1 during the logarithmic growth phase. After 24 h, cell proliferation was detected using fluorescence microscopy according to EdU (RiboBio, Guangzhou, China).^26^

GMEC apoptosis was detected by flow cytometry. These cells were transfected for 24 h in 6-well plates, including miR-2284b mimics/miR-2284b inhibitor and NC/inhibitor NC. After 24 h, corresponding reagents were added according to the Annexin V-FITC Apoptosis Detection Kit protocol (Solarbio, CA1020), and the cell apoptosis rate was analyzed by flow cytometry.

### ELISA analysis

These cells were transfected for 24 h in 6-well plates, including miR-2284b mimics/miR-2284b inhibitor and NC/inhibitor NC. Twenty-four hours later, the cell supernatant was collected according to the ELISA kit protocol, and finally, the absorption intensity of light at 450 nm wavelength was detected by an enzyme labeling analyzer.

### Construction of a CSN1S1 overexpression vector and CHECK2 vector

According to a National Center for Biotechnology Information (https://www.ncbi.nlm.nih.gov/) site search of the CSN1S1 gene 3’UTR and CDS region sequence and TargetScan (http://www.targetscan.org/) website prediction of the combination of miR-2284b and CSN1S1 gene loci an appropriate length of the sequence was chosen, and PCR amplification primers were designed. The target fragment was amplified using reverse-transcribed cDNA as the template, and the recovered fragment was connected to the T vector. The receptive cells were used to expand the culture, and the CSN1S1 gene fragment connected to the T vector was recovered in the medium. The CSN1S1 gene fragment connected to the T vector was cleaved by the corresponding enzyme, and the target fragment was purified and recovered. It was then attached to the CHECK2 vector. During the construction of the overexpression vector, the amplified product was connected to the T vector, expanded culture using the receptor cells, and then double digested and connected to the pc DNA 3.1 vector.

### Double luciferase activity detection

293T cells were plated in 48-well plates. During the logarithmic growth phase, CHECK2-CSN1S1, miR-2284b mimic/inhibitor, and NC/inhibitor NC were transfected, and the amount of transfection reagent was decreased according to the specification ratio of Lipofectamine^TM^ RNAiMAX (Invitrogen protocol). The activity of double luciferase was detected after 36 h.

### Statistical analysis

All data of this study was calculated by SPSS 26.0 (SPSS Inc., Chicago, IL, USA). One-way ANOVA and T-test were used to compare the differences. The data were presented as the mean ± standard deviation. The differences were considered significant at *P* < 0.05 and extremely significant at *P* < 0.01.

## Results

### Effects of miR-2284b on the proliferation of GMECs

First, four mimics, miR-2284b mimic/inhibitor, NC, and inhibitor NC (from Suzhou Genepharma Co., Ltd.), were synthesized and transfected into 6-well plates of GMECs. After 24 h, RNA was extracted from the cells for reverse transcription. Finally, the transfection efficiency of the simulant was detected by RT–qPCR. The results showed that compared with the NC group, the expression of miR-2284b in cells transfected with miR-228b mimics was significantly increased (P < 0.01). Compared with the inhibitor NC group, the expression of miR-2284b in cells transfected with miR-2284b inhibitor was significantly decreased (P < 0.05). The results showed that miR-2284b mimics and miR-2284b inhibitor were effectively expressed in cell transfection (Supplementary Figure 1).

**Figure 1.**
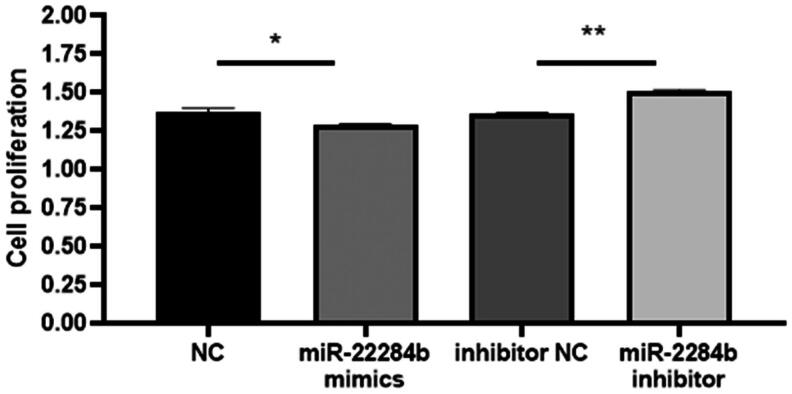
MiR-2284b Inhibited cell proliferation in GMECs. Each group of experiments was repeated three times (mean ± SD). * means *P* < 0.05; ** means *P* < 0.01.

Then, logarithmic growth cells were cultured in 96-well plates and transfected with four mimics, miR-2284b mimic/inhibitor, NC, and inhibitor NC. After 24 h, cell viability was detected with a CCK8 kit. The results showed that transfected miR-2284b cells significantly inhibited cell viability compared with the NC group (P < 0.05), and miR-2284b transfected into inhibitor cells significantly restored the inhibitory effect of miRNAs on cell viability compared with inhibitor NC cells (P < 0.01) (Figure 1).

### Effect of miR-2284b on apoptosis of GMECs

**Figure 2.**
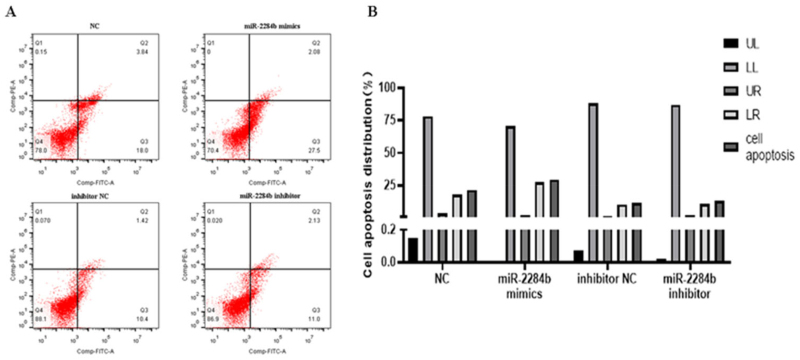
miR-2284b Promoted GMECs apoptosis. A shows the image after flow cytometry, B the analysis of cell apoptosis data. The lower left quadrant (LL) showed normal cells. The upper left quadrant (UL) showed necrotic cells. The upper right quadrant (UR) showed late apoptotic cells. The lower right quadrant (LR) showed early apoptotic cells.

Four mimics, miR-2284b mimic/inhibitor, NC and inhibitor NC, were transfected into GMECs cultured in six-well plates. After 24 h, they were treated with an apoptosis kit and stained with Annexin V-FITC/PI. The number of apoptotic cells was detected by flow cytometry. The results showed that the apoptosis rate of the NC group was 21.84% and that of the experimental group transfected with miR-2284b was 29.58%. Compared with the NC group, the number of apoptotic cells with high expression of miR-2284b increased by 7.74%. In the other group of cells transfected with the inhibitor of miR-2284b and the experimental group transfected with the inhibitor of NC, the cell apoptosis rates were 13.31% and 11.82%, respectively. Although the apoptosis rate was slightly increased after the low expression of miR-2284b, it was basically the same as the control group. There was no significant effect on apoptosis (Figure 2).

### Effects of miR-2284b on the lactation of GMECs

αs1-casein and αs2-casein are the milk proteins in goat milk that easily cause infant allergy, and β-casein and κ-casein are important milk component detection indices reflecting the protein content in goat milk. Four mimics, miR-2284b mimic/inhibitor, NC, and inhibitor NC, were transfected into GMECs cultured in six-well plates. After 48 h, the secretion of GMECs was detected by ELISA kits for four caseins.

**Figure 3.**
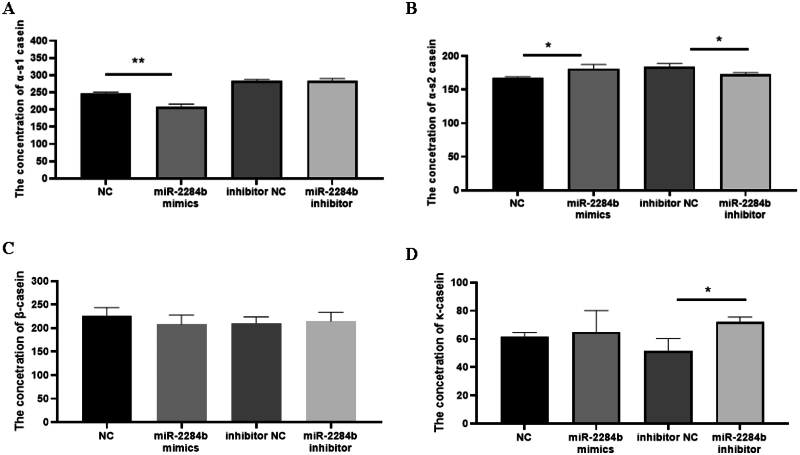
MiR-2284b Regulates the secretion of GMECs casein. Figure A, B, C, and D respectively show the content of four casein proteins in GMECs by using ELASA method. Each group of experiments was repeated three times (mean ± SD). *means *P* < 0.05; **means *P* < 0.01.

The results showed that compared with the NC group, the content of αs1-casein secreted by miR-2284b-transfected cells was significantly decreased (P < 0.01), and the secreted αs2-casein content significantly increased (P < 0.05) but had no effect on β-casein and κ-casein contents. Compared with the inhibitor NC group, the cells transfected with the miR-2284b inhibitor secreted αs1-casein, and the β-casein content did not change significantly. However, αs2-casein content decreased significantly (P < 0.05), and κ-casein content was significantly increased (P < 0.05) (Figure 3).

### Two new sequences of the CSN1S1 gene were obtained by sequencing

According to the National Center for Biotechnology Information (https://www.ncbi.nlm.nih.gov/) for the CSN1S1 gene variant 1 CDS, primers were designed. The CDS region of the CSN1S1 gene was amplified. The results showed that there were two different sequences (Supplementary Table 1).

### miR-2284b downregulates CSN1S1 expression in GMECs

TargetScan (http://www.targetscan.org/) was used to predict potential target genes of miR-2284b and identified a CSN1S1-3′UTR sequence of 60-67 bp with miRNA binding sites. The wild-type CSN1S1 vector CSN1S1-3’UTR CHECK 2 double luciferase reporter vector was constructed. miR-2284b mimic/inhibitor, NC, inhibitor NC and the CHECK 2 vector were cotransfected into GMECs, and luciferase activity was detected by a kit 24 h later.

The results showed that the luciferase activity was significantly decreased compared with the miR-2284b corouted cell group and the corouted NC group (P < 0.01). There was no significant difference in luciferase activity between the cotransferred miR-2284b inhibitor group and the cotransferred miR-2284b inhibitor NC group. CSN1S1 was indeed the target gene of miR-2284b (Supplementary Figure 2).

**Figure 4.**
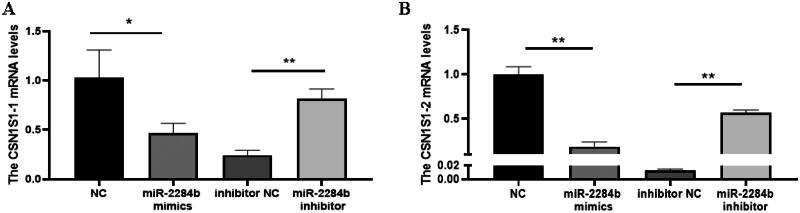
MiR-2284b Specifically targets CSN1S1. Figure A and B respectively show the CSN1S1-1 and CSN1S1-2 mRNA expression in GMECs. Each group of experiments was repeated three times (mean ± SD). *means *P* < 0.05; **means *P* < 0.01.

Subsequently, the regulatory effect of miR-2284b on CSN1S1 in GMECs was further verified. The cells were placed in six-well plates and transfected into miR-2284b mimic/inhibitor, NC and inhibitor NC during the logarithmic growth phase. RNA was extracted 24 h later, and the mRNA levels of two CSN1S1 genes were detected by RT–qPCR after reverse transcription. The results showed that the mRNA level of the CSN1S1-1 gene was significantly decreased in the miR-2284b-transfected cells compared with the NC group (P < 0.05), and the mRNA level of CSN1S1-2 was significantly decreased (P < 0.01). The mRNA levels of both the CSN1S1-1 and CSN1S1-2 genes were significantly increased in cells transfected with the miR-2284b inhibitor compared with the inhibitor NC group (P < 0.01). This proved that miR-2284b had regulatory effects on both the CSN1S1-1 and CSN1S1-2 genes (Figure 4).

### Transfection efficiency of the CSN1S1 gene

To further investigate the regulatory effect of the CSN1S1 gene on α-s1 casein, the overexpression vectors pcDNA3.1-CSN1S1-1 (pc-CSN1S1-1) and pcDNA3.1-CSN1S1-2 (pc-CSN1S1-2) were constructed. Interference RNA (si-CSN1S1) was designed to target the common part of the two gene sequences.

**Figure 5.**
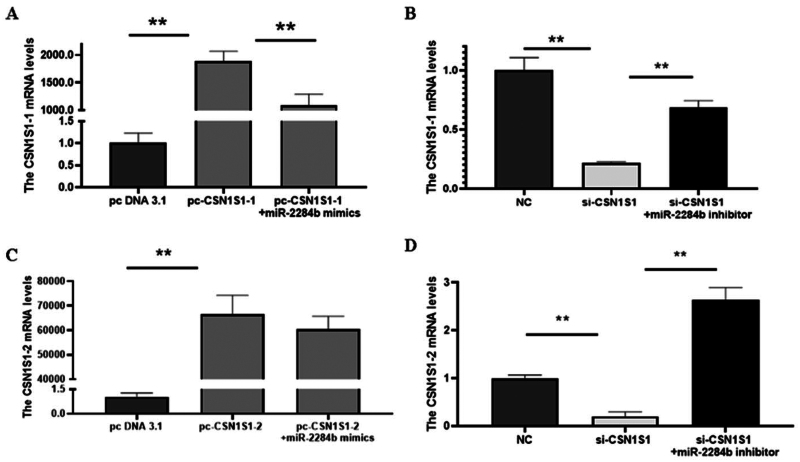
Efficiency detection of CSN1S1 overexpression and interference. A and C are the transfection efficiency of CSN1S1-1/-2 overexpression, and B and D are the transfection efficiency of CSN1S1-1/-2 interference. Each group of experiments was repeated three times (mean ± SD). *means *P* < 0.05; **means *P* < 0.01.

The results showed that compared with the NC group and pc-CSN1S1-1 + miR-2284b mimic group, the mRNA level of CSN1S1-1 was significantly increased (P < 0.01), and the mRNA levels of CSN1S1-1 were significantly decreased in cells transfected with si-CSN1S1 compared with the inhibitor NC group and si-CSN1S1 + miR-2284b inhibitor group (P < 0.01). This proved that pcDNA3.1-CSN1S1-1 and si-CSN1S1 were effective on the CSN1S1-1 gene in GMECs. The mRNA level of CSN1S1-2 was significantly increased in pcDNA3.1-CSN1S1-2 cells transfected with PCDNA3.1-CSN1S1-2 compared with the NC group (P < 0.01). Compared with the pc-CSN1S1-2 + miR-2284b mimic group, the mRNA level of CSN1S1-2 did not change. The mRNA levels of CSN1S1-2 were significantly decreased in cells transfected with si-CSN1S1 compared with the inhibitor NC and si-CSN1S1 + miR-2284b inhibitor (P < 0.01). This proved that pcDNA3.1-CSN1S1-2 and si-CSN1S1 were effective on the CSN1S1-2 gene in GMECs (Figure 5).

### Effects of the CSN1S1 gene on the proliferation of GMECs

**Figure 6.**
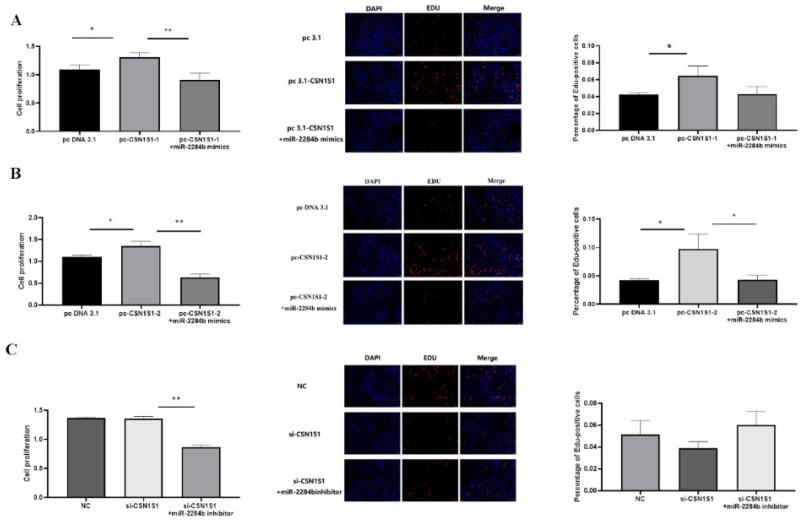
CSN1S1-1/-2 Promote the proliferation of GMECs. A shows the results of cell proliferation using CCK8 and EDU methods after overexpression of CSN1S1-1, B shows the results of cell proliferation using CCK8 and EDU methods after overexpression of CSN1S1-2, C shows the results of using CCK8 and EDU methods after interfering with CSN1S1 cell proliferation results. Each group of experiments was repeated three times (mean ± SD). *means *P* < 0.05; **means *P* < 0.01.

The results showed that PC-CSN1S1-1 significantly increased cell proliferation compared with pcDNA3.1 (P < 0.05). Compared with pc-CSN1S1-1, pc-CSN1S1-1 + miR-2284b mimics significantly inhibited the proliferation effect of the CSN1S1-1 gene on cells in the EdU experiment but had no significant difference in the proliferation effect in the CCK8 experiment. Compared with pcDNA3.1, PC-CSN1S1-2 significantly increased cell proliferation (P < 0.05). pc-CSN1S1-2 + miR-2284b mimics significantly inhibited the proliferation of the CSNIS1-2 gene in the CCK8 assay compared with pc-CSN1S1-1 (P < 0.05), and the results were significant in the EdU experiment (P < 0.01). There was no significant difference between the results of the si-CSN1S1 and NC groups, and there was no significant difference between the results of the si-CSN1S1 + miR-2284b inhibitor and si-CSN1S1 in the EdU experiment. However, in the CCK8 experiment, the results showed that the si-CSN1S1 + miR-2284b inhibitor group significantly decreased the cell proliferation level compared with the si-CSN1S1 group (P < 0.01) (Figure 6).

### Effect of the CSN1S1 gene on apoptosis of GMECs

**Figure 7.**
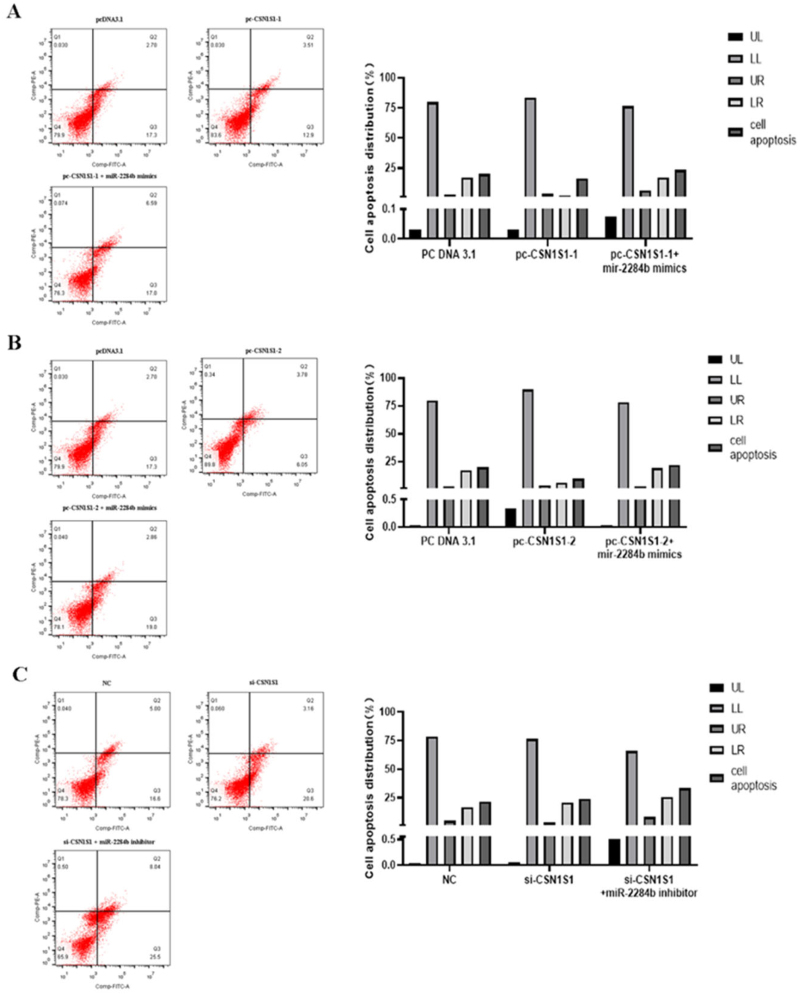
CSN1S1-1/-2 Genes inhibit the apoptosis of GMECs. A shows the experimental results of overexpression of the CSN1S1-1, B shows the experimental results of the overexpression of the CSN1S1-2, C shows the experimental results of the si-CSN1S1 gene. The lower left quadrant (LL) showed normal cells. The upper left quadrant (UL) showed necrotic cells. The upper right quadrant (UR) showed late apoptotic cells. The lower right quadrant (LR) showed early apoptotic cells.

The results showed that the apoptosis rate was 20% in the pcDNA3.1 group, 16.41% in the experimental group transfected with pc-CSN1S1-1, and 9.83% in the experimental group transfected with pc-CSN1S1-2. The high expression of CSN1S1-1 reduced the apoptosis rate by 3.59%. The high expression of CSN1S1-2 decreased the apoptosis rate by 10.17%. The apoptosis rate of the pc-CSN1S1-1 + miR-2284b mimic group was 23.59% and 7.18% higher than that of the pc-CSN1S1-1 group and 21.86% higher than that of the pc-CSN1S1-1 + miR-2284b mimic group. CSN1S1 promotes apoptosis. The apoptosis rate was 21.6% in the NC group and 23.76% in the si-CSN1S1-transfected group, which increased by 2.16%. The apoptosis rate of the experimental group transfected with si-CSN1S1 + miR-2284b inhibitor was 36.47%, which was increased by 12.71% compared with the si-CSN1S1 group (Figure 7).

### Effects of the CSN1S1 gene on casein synthesis in GMECs

**Figure 8.**
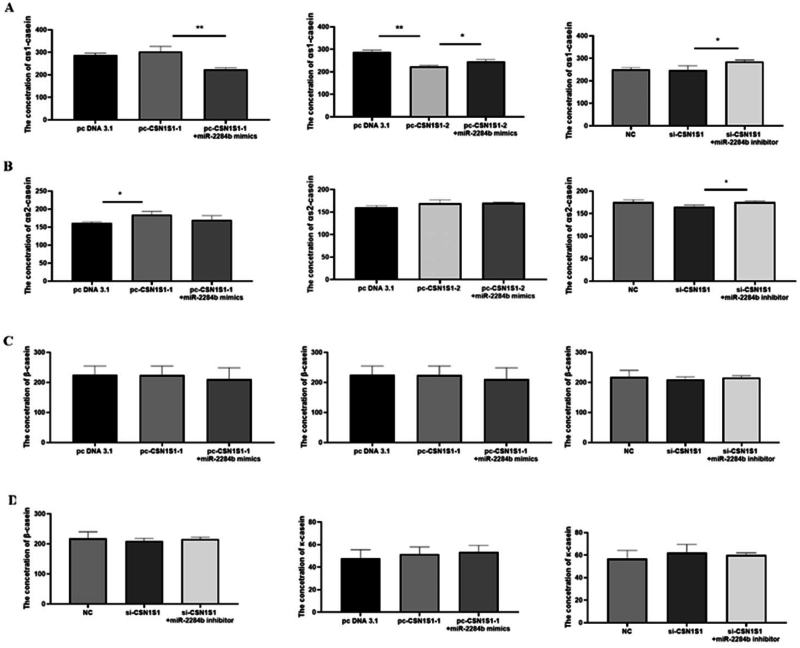
The effect of CSN1S1 on the content of various caseins in GMECs. 48h after transfection, ELISA method was used to detect the amount of various caseins secreted by GMECs. Figure a detects αs1-casein, figure B detects αs2-casein, figure C detects β-casein, and figure D detects κ-casein. Each group of experiments was repeated three times (mean ± SD). *means P < 0.05; **means P < 0.01.

The results showed that, compared with the group transfected with pc DNA 3.1, the experimental group transfected with PC-Csn1s1-only αs2-casein content was significantly increased (P < 0.05), and the contents of αs1-casein, β-casein and κ-casein had no significant difference. Compared with the pc-CSN1S1-1 transfected group, the experimental group transfected with pc-CSN1S1-1 + miR-2284b mimics had significantly lower αs1-casein content (P < 0.01), and the contents of αs2-casein, β-casein and κ-casein had no significant difference. Compared with the group transfected with pcDNA3.1, the experimental group transfected with pc-CSN1S1-2 had significantly lower αs1-casein content (P < 0.01), and the contents of αs2-casein, β-casein and κ-casein had no significant difference. Compared with the pc-CSN1S1-2 transfected group, the experimental group transfected with pc-CSN1S1-2 + miR-2284b mimics had significantly higher αs1-casein content (P < 0.05), and the contents of αs2-casein, β-casein and κ-casein had no significant difference. There was no significant difference in casein content between the transfected NC cells and the transfected si-CSN1S1 cells. Compared with the transfected si-CSN1S1 + miR-2284b inhibitor group, the contents of αs1-casein and αs2-casein in the transfected si-CSN1S1+ miR-2284b inhibitor cells were significantly increased (P < 0.05), and β-casein and κ-casein contents had no significant difference (Figure 8).

## Discussion

MiRNAs are a class of short noncoding ribonucleotides that play a role in the posttranscriptional regulation of gene expression. They are powerful regulators of various cellular activities, including the regulation of cell growth, differentiation, development and apoptosis.^27^ Therefore, there have been many studies on the role of miRNAs in mammary gland development, lactation and regulation of mammary epithelial cells. In 2007, Gu ZL first identified 59 miRNAs by sequencing in the mammary glands of dairy cows, suggesting that some of them may play a specific role in the growth and development of the mammary gland.^28^ Ibarra then discovered specific expression patterns of these miRNAs in self-renewing progenitor cells in the mouse mammary gland and that these signals could be used to increase or decrease the corresponding stem cell population.^29^ This study is the first to clarify the link between miRNAs and breast cells. In recent years, an increasing number of detailed studies on the regulatory mechanism of miRNAs have been published. For example, miR-93-5p, miR-743a-3p and miR-182-5p can promote apoptosis of mouse mammary epithelial cells, while miR-1195 and miR-181b-5p inhibit apoptosis;^30^ miR-497 inhibits the production of triglycerides and unsaturated fatty acids in bovine mammary epithelial cells;^31^ and miR193a-5p regulates polyunsaturated fatty acid metabolism in bovine mammary epithelial cells by targeting FADS1.^32^

There is less research on the entire family of miR-2284, which is a relatively new family of miRNAs. The miR-2284 family was first discovered in 2013 and is most likely endemic to ruminants such as cattle and goats.^33^ It was found that the differential expression of the miR-2284 family was mainly concentrated in the lactation and nonlactation periods of ruminants,^23^ which suggests that the miR-2284 family may be related to mammary gland development and lactation. In the early stage of this study, miRNAs in the mammary glands of dairy goats in early lactation and peak lactation were sequenced, and it was found that the expression level of miR-2284b was different during the two periods, so it was speculated that it might have a regulatory effect on mammary gland development and lactation of dairy goats. In the experiment, miR-2284b mimic/inhibitor, NC and inhibitor NC were transfected into GMECs to study the regulatory effects of miR-2284b mimic/inhibitor, NC and inhibitor NC on cell proliferation, apoptosis and casein secretion in milk. The results showed that miR-2284b inhibited the proliferation of GMECs, promoted cell apoptosis, inhibited the secretion of αs1-casein and promoted the secretion of αs2-casein by mammary epithelial cells but had no regulatory effect on β-casein and κ-casein. These experimental results all suggest that miR-2284b regulates casein secretion in milk by regulating the activity of GMECs. MiR-2284b can be considered a factor affecting mammary gland development, lactation and milk quality in dairy goats.

Then, miR-2284b can regulate the proliferation and apoptosis of GMECs as well as the synthesis and secretion of casein in milk. However, the specific regulatory mechanism of miR-2284b is still unclear and needs to be further explored in experiments. It was found that miRNAs function and recognize the target mainly through the interaction between the seed sequence of miRNAs (2–8 nt) and the 3′-untranslated region (3′-UTR) of the target mRNA, and these 7–8 nt mediate most of the inhibition of miRNAs.^34^

Through high-throughput sequencing in the laboratory, it was found that miR-2284b was differentially expressed in the mammary glands of dairy goats in the early and peak lactation periods. Therefore, it is speculated that miR-2284b may interact with casein-encoding genes. The target binding site between miR-2284b and the CSN1S1 gene was predicted by a bioinformatics website. It is speculated that miR-2284b may affect the synthesis and secretion of casein in GMECs by targeting the CSN1S1 gene.

This study first verified that the 3’UTR of CSN1S1 could indeed bind to miR-2284b through the dual luciferase reporting system, confirming that CSN1S1 was the target gene of miR-2284b. Then, the regulation of miR-2284b on the CSN1S1 gene was verified by detecting the expression level of CSN1S1-1/-2 mRNA and the secretion of αs1-casein. MiR-2284b can inhibit the expression of the CSN1S1 gene and the secretion of αs1-casein in GMECs. The experimental results of the coexpression of miR-2284b and CSN1S1-1/-2 also supported this conclusion. In most cases, miR-2284b can inhibit the effect of CSN1S1-1/-2 overexpression on GMECs, and similar results can be observed in the regulation of cell proliferation, apoptosis and casein secretion. miR-2284b can negatively regulate the expression of CSN1S1-1/-2 and thus regulate the synthesis of αs1-casein in GMECs.

Milk composition affects the nutritional value, technological characteristics and quality of dairy products.^35^ There are four types of casein: αS1 (αs1CN), β (β-CN) and αS2 (αs2-CN), which are calcium-sensitive proteins, and κ-casein, which plays an important role in maintaining the micellar stability of casein.^36^ The genes that encode casein seem to have been evolving rapidly.^37^ They are highly polymorphic in almost all species. At the same time, some sequence variation has occurred in the upstream and downstream gene regions encoding casein, which is also suspected to affect the expression of casein genes and the contents and proportions of these casein proteins in goat milk.^38^ In 2021, Siham sequenced 18 more casein gene variants in the Saanen dairy goat, bezoar ibex and alpine ibex, of which 6 were αs1-casein. In the six new DNA sequences, there were both amino acid substitutions and new proteins.^39^

In this study, two unreported CSN1S1 gene sequences were detected, and the base deletion and mutation were located in the CDS region of the gene, which resulted in both synonymous substitution at the amino acid level and the emergence of new amino acids. Serine (S) and glutamic acid (E) appeared in CSN1S1-1, and arginine (R) was replaced by lysine (K). The original position of CSN1S1-2 was cysteine (C) and glutamine (Q). The expression of the two CSN1S1 genes in GMECs is also different. Although overexpression of the CSN1S1-1/-2 gene can significantly promote the proliferation of GMECs and inhibit the apoptosis of GMECs, overall, the growth regulation effect of the CSN1S1-2 gene on GMECs is better than that of the CSN1S1-1 gene. This may be due to differences in the amino acids translated between the two. In terms of casein regulation, CSN1S1-1 did not affect the content of αs1-casein in GMECs but significantly upregulated the content of αs2-casein in GMECs. However, CSN1S1-2 downregulated the content of αs1-casein in GMECs. It is possible that these two CSN1S1 variants may inhibit casein production in GMECs by affecting other pathways regulating αs1-casein metabolism.

## Conclusion

This study mainly explored the mechanism by which miR-2284b regulates α-s1 casein synthesis in GMECs. First, it was demonstrated that miR-2284b inhibited the proliferation of GMECs and promoted apoptosis. MiR-2284b inhibited the synthesis of αs1-casein and promoted the synthesis of αs2-casein in GMECs but had no effect on the synthesis of β-casein and κ-casein. Therefore, miR-2284b can be considered a factor affecting mammary gland development, lactation and milk quality in dairy goats. Then, bioinformatics analysis revealed two new sequences of the CSN1S1 gene. Moreover, miR-2284b binds to the 3’UTR of the CSN1S1 gene and negatively regulates the mRNA expression of CSN1S1-1/-2 and the production of αs1-casein. CSN1S1-1/-2 promoted the proliferation and inhibited the apoptosis of GMECs, and the regulatory effect of CSN1S1-2 was better than that of CSN1S1-1. MiR-2284b negatively regulated proliferation and apoptosis through CSN1S1-1/-2. CSN1S1-1/-2 did not regulate the synthesis of αs1-casein in GMECs. Research on the molecular mechanism can provide theoretical guidance for the breeding of dairy goats.

## Supplementary Material

Supplemental Material

## Data Availability

This study was conducted according to the guidelines of the Institutional Animal Care and Use Committee of the School of the Northwest A&F University Animal Experiments Ethics Committee.
