# Supplementary figures and images for: MiR-2284b regulation of α-s1 casein synthesis in mammary epithelial cells of dairy goats
Source: Anim Biotechnol. 2024 Apr 16;35(1):2334725. doi: 10.1080/10495398.2024.2334725 (PMC12674344; doi:10.1080/10495398.2024.2334725)

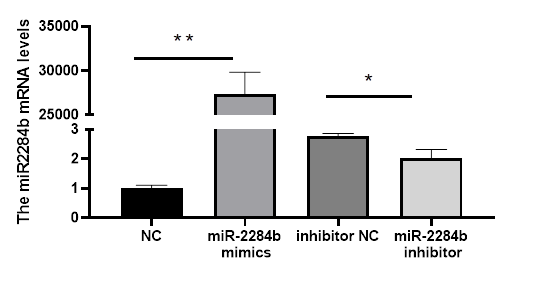

Supplement: Supplemental Material [file LABT_A_2334725_SM5287.zip › supplementary materials/Supplementary Figure 1 Transfection efficiency of miR-2284b.png]

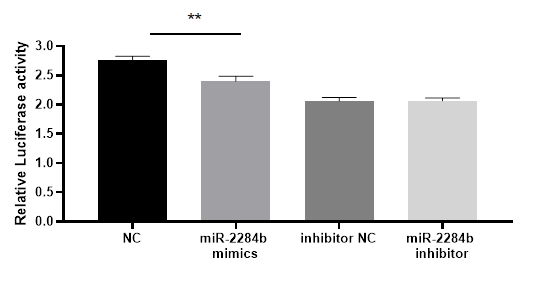

Supplement: Supplemental Material [file LABT_A_2334725_SM5287.zip › supplementary materials/Supplementary Figure 2 The detection of relative luciferase activity.png]
